# Gastrointestinal Survivability of a BSH-Positive *Lacticaseibacillus rhamnosus* VB4 Strain and Its Effect on Bile Acid Deconjugation in a Dynamic In Vitro Gut Model

**DOI:** 10.3390/nu17193179

**Published:** 2025-10-08

**Authors:** Amanda Vaccalluzzo, Gianluigi Agolino, Alessandra Pino, Marianna Cristofolini, Davide Tagliazucchi, Alice Cattivelli, Cinzia Caggia, Lisa Solieri, Cinzia Lucia Randazzo

**Affiliations:** 1 Department of Agriculture, Food and Environment, University of Catania, 95123 Catania, Italy; amanda.vaccalluzzo@unict.it (A.V.); gianluigi.agolino@phd.unict.it (G.A.); alessandra.pino@unict.it (A.P.); ccaggia@unict.it (C.C.); 2 ProBioEtna SRL, Spin Off of the University of Catania, Santa Sofia Street, 100, 95123 Catania, Italy; 3 Department of Life Science, University of Modena and Reggio Emilia, 42122 Reggio Emilia, Italy; marianna.cristofolini@unimore.it (M.C.); davide.tagliazucchi@unimore.it (D.T.); alice.cattivelli@unimore.it (A.C.); lisa.solieri@unimore.it (L.S.)

**Keywords:** bile salt hydrolase, bile acids, dynamic simulator, strain survivability, *bsh* gene expression, metabolomic profile

## Abstract

Background: Bile salt hydrolase (BSH) is a key probiotic trait, as it facilitates both host metabolism and bacterial survival into the gastrointestinal tract (GIT), through bile acid (BA) deconjugation, keeping intestinal homeostasis. Objectives: The present study aims to investigate the viability of the *Lacticaseibacillus rhamnosus* VB4 strain and its effects on bile acid deconjugation during the gastrointestinal tract (GIT) passage, under a fed condition, using the in vitro SHIME^®^ (Simulator of the Human Intestinal Microbial Ecosystem) model. Methods: Gastric, small intestinal and colonic fractions were monitored and a fecal slurry from a healthy donor was inoculated into the colonic compartment to establish the intestinal microbiota. Samples were collected at the end of stomach, duodenum, jejunum, ileum phases, and colon after 0, 16 and 24 h. Strain survival was assessed by culturing method, and *bsh* gene expression was revealed by quantitative PCR (qPCR). In addition, UHPLC/HR-MS was performed to reveal the hypothetical changes in BAs profile after strain administration. Results: Good survivability of the VB4 strain in the upper GIT was revealed. Furthermore, VB4-inculated sample showed sustained expression of *bsh* in both the stomach/small intestine and colon fractions at all sampling times. Analysis of the BAs profile shown that the VB4 strain reduced the levels of the main conjugated BAs in the small intestine under fed condition and improved the deconjugation efficiency during colonic transit compared with the control. Conclusions: These findings highlight the survivability of *L. rhamnosus* VB4 strain inside the gut and its potential as biotherapeutic BAs-mediator candidate, demonstrating that transcriptomic and metabolomic approaches coupled to a dynamic in vitro gut model represent a robust tool for selection of a BSH-positive probiotic candidate.

## 1. Introduction

Lactobacilli, as natural inhabitant of the gastrointestinal tract (GIT), have attracted considerable attention for their probiotic trait and for the capacity to synthesize the bile salt hydrolase (BSH) [1]. BSH is a gut microbial enzyme closely related to bile acids (BAs) metabolism, which catalyzes the first reaction of conjugated BAs hydrolysis, necessary for their biotransformation into deconjugated ones. Gastrointestinal bacteria play a crucial role in the recovery of BAs, acting as molecules with endocrine and paracrine signals, involved in intestinal absorption of dietary and endogenous-derived lipids, as well as glucose metabolism. Primary BAs are synthesized in the liver, and subsequently bond glycine or taurine, thus forming conjugated BAs. After being released into the small intestine, BSH enzyme cleaves the amide bond of glyco- and tauro-conjugated BAs, forming free amino acids and deconjugated BAs, which reaching the colon as secondary BAs through further bioconversion. As suggested, BSH ability is directly implicated in different metabolic pathways, including its role on endogenous cholesterol metabolism [1,2,3,4]. Since free BAs are less soluble and are reabsorbed less efficiently in the ileum, their fecal excretion increases, which in turn stimulates the liver to convert endogenous cholesterol into new BAs. This compensatory mechanism reduces the pool of circulating cholesterol, thus explaining the role of BSH in endogenous cholesterol metabolism [2]. However, the full mechanisms underlying the complex interaction between BAs and cholesterol metabolisms remain poorly known [3]. The activity of BSH was further validated by the presence of genes encoding the BSH enzyme in the genome of intestinal bacteria. This activity could further support the survivability of BSH-positive microorganisms and improve their colonization within the intestine. In this regard, studies on *bsh* mutants showed growth deficiency by in vitro testing, suggesting the involvement of *bsh* genes in bile tolerance [5,6,7]. Taxonomic study on *bsh* gene distribution distinguish different phylotype, highlighting different copy number of the gene across *Lactobacillus* genera, as well as the synthesis of several BSH variant with different functional properties [8,9,10]. Given the pivotal impact of BSH activity on bacterial survival gut microbiota, *bsh* gene detection in lactobacilli, has been recognized as a functional biomarker for probiotic selection [8].

More recently, research is focused on the use of predictive methods, such as SHIME^®^ system, often integrated with high-throughput technologies, to quantify and characterize cells and molecules involved in the microbial ecosystem. These strategies are applied to detect functional properties arising from the interaction between probiotic supplementation and the gut microbiota. At this regard, in vitro studies conducted using the Simulator of the Human Microbial Ecosystem (SHIME^®^, ProDigest, Belgium) are considered a viable strategy to better understand the effect of potential probiotic strains and to make them a more suitable product for the formulation of dietary and/or pharmaceutical supplements [11]. The SHIME^®^ is a GIT model, which mimics the dynamic and physiological processes starting from the stomach until the colonic fractions, in response to a probiotic or prebiotic action [12,13]. Therefore, in this work, the upper GIT SHIME^®^ model, extended to the colonic fraction, was used to assess the survival and BAs deconjugation capacity of the BSH-positive *Lacticaseibacillus rhamnosus* VB4 strain, previously selected according to Agolino and co-workers [14]. Briefly, the strain demonstrated BSH activity, by in vitro test under simulated gastrointestinal conditions, and the expression putative *bsh* genes, by transcriptional profile analysis. In particular, by combining transcriptomic and metabolomic approaches, we sought to better understand the action of BSH by evaluating a dynamic process of BAs colonization and conjugation, monitoring its passage from the upper part of the GIT to the colonic fraction.

## 2. Material and Methods

### 2.1. BSH-Positive L. rhamnosus VB4 Strain

The *Lacticaseibacillus rhamnosus* VB4 strain, belonging to the microbial collection of ProBioEtna srl, Spin Off of the University of Catania (Catania, Italy), was selected for its probiotic properties and high ability to deconjugate BAs, in accordance with the results obtained in the previous work conducted by Agolino and co-workers [14]. The VB4 strain was freeze-dried by SACCO Srl (Cadorago, Italy) and, prior to inoculation into SHIME^®^ system, its viability was verified using plate culture method with Lactobacilli MRS agar medium (BD Difco^TM^, Milan, Italy). The freeze-dried strain was directly inoculated into the stomach/small intestine reactors and the starting cell density of the VB4 strain was at 9 log_10_ CFU/g.

### 2.2. SHIME^®^ Experiment Set-Up

The dynamic SHIME^®^ model (ProDigest, Ghent, Belgium) was set up under feeding condition according to the protocols proposed by Marzorati and co-workers [15] and Jannin and collaborators [16] to mimic the incubation of stomach, small intestine (St/SI) and colon (C) compartments, using two double-jacketed reactors serially connected, maintained at 37 °C under constant agitation. Both reactors, St/SI and C were maintained under anaerobic conditions, by controlled N_2_, in accordance with the requirements of the nitrogen flushing system. The lyophilized strain was inoculated with an initial cell density of 9 log_10_ CFU/g in the first reactor (St/SI), simulating the digestion of the stomach and small intestine. In detail, under fed condition, a gastric solution (76 mL), containing SHIME^®^ nutritional medium (PDNM001B 20.53 g/L, ProDigest), NaCl (3.63 g/L), KCl (0.65 g/L), 0.4 mL lecithin (13.5 g/L), and 3.6 mL pepsin (40 g/L) at pH 4.6 was added to the vessel and maintained for 120 min with a sigmoidal pH decrease from 4.6 to 2. After incubation in the stomach, a small intestine phase, comprising the duodenum, jejunum, and ileum, was performed. In accordance with published SHIME^®^ protocols [15,16], BAs were supplemented as Oxgall (Difco, bovine bile extract) in the duodenal phase together with pancreatic juice solution, in order to reproduce the physiological presence of BAs in the small intestine. No bile salts were added in the gastric phase. Specifically, during the duodenal phase, a pancreatic juice composition (NaHCO_3_ 7.7 g/L, Oxgall 15 g/L, and pancreatin 10 g/L, added with 2.15 mL trypsin 10 g/L and 2.7 mL chymotrypsin 10 g/L) was added. The pH of the small intestine was gradually increased from 2.0 to 6.5 and maintained at this pH over 27 min to simulate the duodenal fraction, followed by jejunal (pH up to 7.5 maintained for 63 min) and ileal (constant pH 7.5 for 90 min) fractions. An increase in pH was achieved by the addition of NaHCO_3_ (4.8 g/L) at 60, 90, and 120 min, mimicking the dilution of the intestinal contents. The increase and decrease in pH were automatically controlled, by pH-meter probe (ProSense, Oosterhout, The Netherlands), and adjusted by the dosage of HCl (0.5 M) and NaOH (0.5 M). All reagents and chemicals used were of analytical grade and were purchased from Merck (Milan, Italy).

### 2.3. Fecal Slurry Preparation and Colonic Incubation

**Figure 1:**
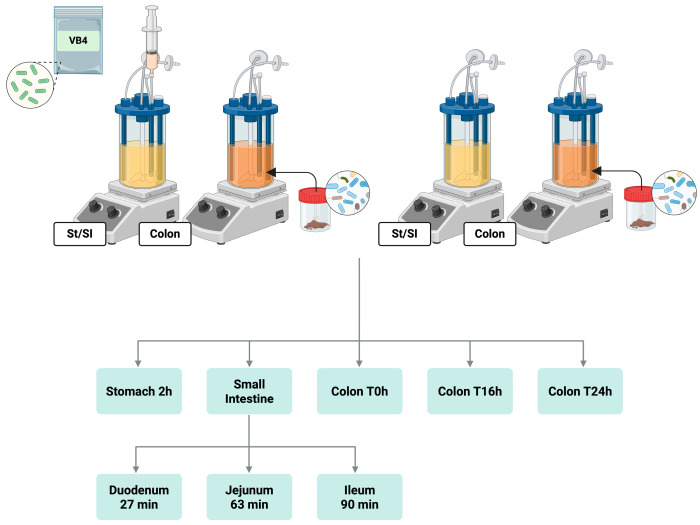
Overview of the SHIME^®^ system setup. The stomach/small intestine (St/SI) reactor connected in series to the colon (C) reactor. The unit was designed for both VB4-inoculated samples (**left**) and control samples (**right**). In addition, a workflow is shown describing the incubation steps and times performed and the collection of intestinal and colon fraction samples. St/SI, stomach/small intestine; VB4, *L. rhamnosus* VB4 strain.

An extension of the colonic incubation was further proposed. The digested solution from the stomach/small intestine reactor was transferred to the second reactor, simulating colonic conditions, and maintained for 24 h. A fecal sample from a healthy donor (male, 50y) was chosen for the SHIME^®^ experiment. The collection of data from the use of the human fecal sample was approved by the Ethics Committee of the University of Modena and Reggio Emilia (CEAR) on 20.01.2025, and informed consent for the experimentation was obtained from the subject involved in the study. The fecal slurry was obtained according to the protocol proposed by Marzorati and collaborators [15] and inoculated into the colonic reactor. In detail, a 1:10 (*w*/*v*) mixture of fecal sample and anaerobic phosphate buffer (K_2_HPO_4_ 8.8 g/L; KH_2_PO_4_ 6.8 g/L; sodium thioglycolate 0.1 g/L; sodium dithionite 0.015 g/L) was homogenized for 10 min (BagMixer 400, Interscience, Louvain-LaNeuve, Belgium), and the mixture was centrifuged (2 min, 500 g) and the large particles were removed. The obtained fecal slurry (20% *v*/*v*) was added to 160 mL fresh colonic anaerobic medium [KH_2_PO_4_ (6.6 g/L), K_2_HPO_4_ (20.5 g/L), NaCl (5 g/L), yeast extract (2 g/L), peptone (2 g/L), glucose (1 g/L), starch (2 g/L), mucin (1 g/L), L-cysteine HCl (0.5 g/L), Tween^®^ 80 (2 mL)], 40 mL of anaerobic PBS [K_2_HPO_4_ (8.8 g/L), KH_2_PO_4_ (6.4 g/L), NaCl (8.5 g/L) and L-cysteine HCl (0.5 g/L)]. In the colonic compartment, a fixed pH range between 6.5 and 5.8 was implemented, adjusted automatically, maintaining an anaerobic condition at 37 °C and an agitation of 90 rpm. A reactor without the addition of the probiotic strain was used as a control sample, following all of the steps of the digestion, from the stomach to the colon compartments. After few minutes of stabilization, aliquots collected from both reactors were subjected to qPCR analysis for Lactobacilli quantification. All the SHIME^®^ assays were performed in duplicate. In the stomach/small intestine reactor, samples were collected at the end of the incubation phases of the stomach, duodenum, jejunum, and ileum, and subjected to culture-dependent and culture-independent analysis. While in the colonic fraction, lumen samples were collected after 0, 16, and 24 h of colonic incubation. All the analyses were assessed in triplicate. All reagents and chemicals used were of analytical grade and were purchased from Merck (Milan, Italy). A schematic representation of the experimental workflow is shown in Figure 1.

### 2.4. Detection of Viability by Culture-Dependent Method

The viability of *L. rhamnosus* VB4 strain was evaluated during GIT passage, from the stomach to ileal phase, by plate count. Briefly, the bacterial count was performed by plating serial ten-fold dilutions using the anaerobic PBS, containing 8.8 g/L of K_2_HPO_4_, 6.4 g/L of KH_2_PO_4_, 8.5 g/L of NaCl, and 0.5 g/L of L-cysteine HCl, then cultured on Lactobacilli MRS Agar medium (BD Difco™) and incubated under anaerobic conditions at 37 °C for 48 h. Colony counts were performed on plates yielding between 30 and 300 colonies. The plate count assays were performed in triplicate and results were reported as mean values log_10_ CFU/mL and standard deviation.

### 2.5. Detection of Gene Expression of BSH-Positive VB4 Strain2

#### 2.5.1. RNA Extraction

All samples collected during the simulation with the SHIME^®^ model were subjected to RNA extraction. Specifically, 2 mL of stomach/intestinal small intestine and lumen samples and 0.3 g of mucosal samples were centrifuged at 20,000× *g* for 10 min, washed twice with anaerobic PBS and subjected to ZymoBIOMICS^TM^ RNA Miniprep Kit (Zymo Research, Orange, CA, USA), with prior mechanical breakage with Precellys Evolution Homogenizer (Bertin Technologies, Montigny-le-Bretonneux, France) at 10,000 rpm for 2 min, repeated three times, interspersed with breaks on ice. The concentration, integrity and quality of RNA templates were checked using the Qubit^TM^ 4 Fluorometer (Thermo Fisher Scientific, San Jose, CA, USA).

#### 2.5.2. RT-PCR and RT-qPCR Assays

The RNA templates were subjected to reverse transcription PCR (RT-PCR) analysis using the QuantiTect Reverse Transcription Kit (Qiagen, Hilden, Germany), according to the manufacturer’s instructions. The complementary DNA (cDNA) obtained was subjected to RT-qPCR with a *L. rhamnosus* species-specific primer pair, designed in the previous study conducted by Agolino and collaborators [14]. In detail, the reaction included 10 µL of QuantiNovaTM SYBR Green PCR Kit (Qiagen), 0.7 µM of each primer Bsh_rha_qF2 (GGAATACGGGTGCATACAA) and Bsh_rha_qR2 (CAGGCCAAACATGCCATAAC), 50 ng of cDNA template and 4.2 µL of Dnase/Rnase-free water (Thermo Fisher Scientific). Cycling conditions comprised a holding phase at 95 °C for 2 min, followed by 40 cycles at 95 °C for 5 s, 60 °C for 10 s, and 60 °C for 30 s. The melting range was set between 60 °C and 95 °C. In addition, according to the protocol proposed by Agolino and co-workers [14], RT-qPCR was also performed for the 16S rRNA housekeeping (*HKG*) gene with *L. rhamnosus*-specific primers 16S_F1 (GTAGGTGGCAAGCGTTATCC), and 16S_Rw1 (GATGCGCTTCCTCGGTTAAG). The reaction mixture and reagent concentrations included 10 µL of QuantiNovaTM SYBR Green PCR Kit (Qiagen), 0.7 µM of each primer. The qPCR cycling parameters include a holding phase at 95 °C for 2 min, followed by 40 cycles at 95 °C for 5 sec and 60 °C for 60 sec. The melting range was set from 60 °C to 95 °C. Specificity and amplification efficiency (E) were performed using a 10× dilution of the microbial gDNA of *L. rhamnosus* VB4 as a reference standard. Slope of the regression curve between the logical values of the cDNA concentrations and the mean values of the cycle threshold (Ct) was used to calculate the primer efficiency using the equation: E= 0.5 (10 (−1/slope)) × 100. A Rotor Gene Q instrument (Qiagen, Milan, Italy) was used to perform the reaction. Each reaction was repeated at least three times.

For both *HSG* and *bsh* genes, standard curves were created by plotting qPCR Ct values against the log inputs genome copy number obtained from standard genomic DNA. The copy numbers determined for DNA standards were calculated using the formula:

$$number\;of\;copies=\frac{amount\;of\;DNA\;\left(ng\right)\times 6.022\times 10^{23}}{length\;of\;the\;genome\;\left(bp\right)\times 1\times 10^{9\;}\times 650)}$$

where length of genome was established in Agolino et al. [14]; Na is the (6.022 × 10^23^ molecules/mol is the Avogadro’s number), 660 g/mol is the average weight of a single base pair, and 10^9^ is the conversion factor. For *bsh*-based RT-qPCR standard curves enabled an estimation of cDNA copy number as *bsh* gene was in single copy. For 16S-based RT-qPCR, a correction was applied to take into account the number of ribosomal RNA operon copies, as established in Agolino et al. [14].

### 2.6. Detection of BAs from SHIME^®^ Samples

The semi-quantitative analysis of individual BAs in samples from the SHIME^®^ model (both in control and VB4-inoculated samples) was carried out as reported in Agolino and collaborators [14] by using a high-resolution mass spectrometer. In detail, samples collected from small intestine fractions, including duodenum, jejunum, and ileum phases, and colonic fraction collected after 16 and 24 h of incubation were subjected to BAs detection. Chromatographic separation was performed with a UHPLC Ultimate 3000 module (Thermo Fisher Scientific) equipped with a C18 column (Acquity UPLC HSS C18 Reversed phase, 2.1 × 100 mm, 1.8 µm particle size, Waters, Milan, Italy). Mass spectrometry analysis was carried out through a Q Exactive Hybrid Quadrupole-Orbitrap Mass Spectrometer (Thermo Fisher Scientific). Mobile phases were water with 0.1% formic acid (mobile phase A) and acetonitrile with 0.1% formic acid (mobile phase B) and the flow rate was 0.3 mL/min. An amount of 10 μL of appropriately diluted sample was injected. The chromatographic and mass spectrometry parameters were fully described in Agolino et al. [14]. The analyzed BAs were glycocholic acid (GCA), taurocholic acid (TCA); glycodeoxycholic acid (GDCA) and taurodeoxycholic acid (TDCA). Preliminary analysis demonstrated that taurochenodeoxycholic acid was not present in this specific Oxgall preparation whereas glycochenodeoxycholic acid was only present in minor amounts. Therefore, these two BAs were not further considered for the subsequent analysis. The relative amount of bile acids was determined by integrating the area under the peak (AUP). AUP values were quantified from the extracted ion chromatograms (EIC) calculated for each mass-to-charge ratio of a specific compound (tolerance ± 5 ppm) using the Genesis algorithm function in the Thermo Xcalibur Quantitative Browser.

### 2.7. Statistical Analysis

All the analyses were performed in technical triplicate, and data are presented as mean values ± standard deviation (SD). Statistical analyses and graph generation were conducted using GraphPad Prism 10 (GraphPad Software, La Jolla, CA, USA). Given the small sample size (*n* = 3), formal tests of normality and variance homogeneity could not be reliably applied. Parametric methods were therefore used under the assumption of approximate normality, considering the robustness of ANOVA to moderate variance differences in balanced designs. Microbiological counts and gene expression were analyzed using ANOVA One-way, by using Tukey’s post-hoc test. Whereas the BAs profile was assessed using an ANOVA Two-way. Followed by Dunnett’s post-hoc. Statistical significance was set at *p*-value < 0.05.

## 3. Results

### 3.1. L. rhamnosus VB4 Strain Viability in Stomach/Small Intestine

**Figure 2:**
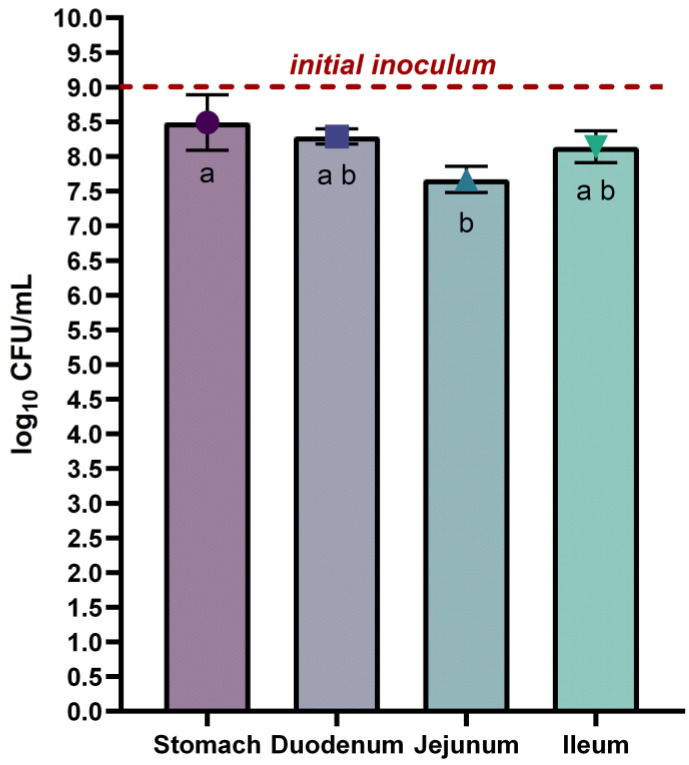
Survival of *Lacticaseibacillus rhamnosus* VB4 in Stomach/Small Intestine compartments. Viable counts were enumerated on agar plate. Data are expressed as average ± SD of the three independent biological replicates. Results are expressed as mean ± SD of log_10_ CFU/mL. Data were analysed using ANOVA One-way with Tukey’s post-hoc test. The horizontal red line indicates the starting cell density of the VB4 strain (9 log_10_ unit). ^a–b^ Different superscript letters indicate significant differences within samples at different GI fractions (at *p* < 0.05).

The *L. rhamnosus* VB4 strain viability in the stomach and small intestine was achieved by plate count and results are displayed in Figure 2. Overall, no substantial differences were detected between the stomach and the small intestine (*p >* 0.05). In detail, the strain exhibited a slight reduction during the final gastric phase, decreasing from the initial inoculum (9 log_10_ CFU/mL) to 8.49 ± 0.40 log_10_ CFU/mL (*p* < 0.05), mainly due to the acidic condition and gastric enzymes of the stomach environment. During the small intestine transit, a progressive decrease of the VB4 cell density was observed from duodenum to jejunum, with values of 8.29 ± 0.11 log_10_ CFU/mL (*p* < 0.05) and 7.67 ± 0.19 log_10_ CFU/mL (*p* < 0.05), respectively. A slight increase in the ileum with a cell density of 8.14 ± 0.23 log_10_ CFU/mL (*p* < 0.05) was achieved.

### 3.2. Bsh Gene Detection Through qPCR Analysis

**Figure 3:**
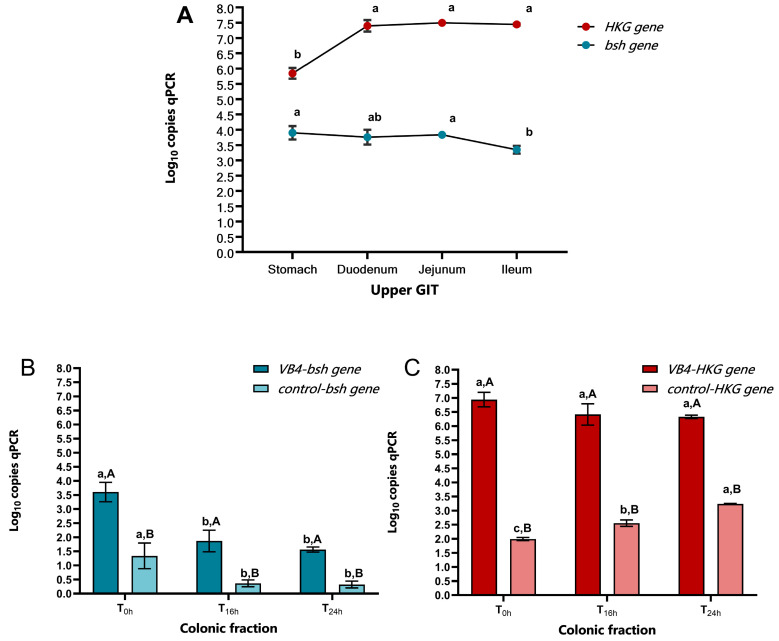
Transcriptional profiles of *bsh* and *HKG* genes in SHIME^®^ samples. (**A**) Gene expression of *bsh* and *HKG* genes in VB4-inoculated sample, along upper GIT. (**B**) *bsh* gene expression in colonic fraction, at 0, 16 and 24 sampling times, of VB4-inoculated and control samples. (**C**) *HKG* gene expression in colonic fraction, at 0, 16 and 24 sampling times, of VB4-inoculated and control samples. Results are expressed as mean ± SD of log_10_ copies qPCR. Data were analysed using ANOVA One-way with Tukey’s post-hoc test. ^a–c^ The different superscript letters indicate significant differences within the same sample over time at *p* < 0.05. ^A–B^ The different superscript letters within the sampling time indicate significant differences between the two treated and control theses at *p <* 0.05. *Bsh*, bile salt hydrolase gene; GIT, gastrointestinal tract; *HKG*, housekeeping gene; qPCR, quantitative PCR; VB4, *L. rhamnosus* VB4 strain.

For the evaluation of the transcriptional profile of the *bsh* gene in different gastrointestinal compartments, gene expression quantification in both the upper gastrointestinal tract and colonic fractions was assessed. The raw qPCR results expressed in ng/µL were converted into absolute copy numbers using an equation based on Avogadro’s constant, the genome length of the reference strain (*L. rhamnosus* VB4) and the intercept of the standard curve. In addition, the housekeeping (*HKG*) gene was also quantified for a more accurate estimate of gene expression capacity. Figure 3 shows the data obtained from the transcriptional test. Specifically, Figure 3A shows significant differences in the *bsh* gene expression profile between the stomach, and ileum phases, with values of 3.90 and 3.35 log_10_ copies qPCR (*p* < 0.05), respectively, and between the jejunum and ileum phases, with a value of 3.83 log_10_ copies qPCR (*p* < 0.05). Conversely, the *HKG* gene was lower in the stomach phase with a value of 5.84 log_10_ copies qPCR (*p* < 0.05), which was significantly different from the duodenum (7.40 log_10_ copies qPCR), jejunum (7.44 log_10_ copies qPCR), and ileum (7.50 log_10_ qPCR copies), which showed a slight increase towards the end of the small intestine.

In Figure 3B, the transcriptional profile of the *bsh* gene of the VB4-inoculated sample in the colonic fraction showed a slight decrease of expression from T_0h_ to T_24h_, with significant differences (*p* < 0.05) of T_16h_ (1.87 log_10_ copies qPCR) and T_24h_ (1.56 log_10_ copies qPCR) compared to T_0h_ (3.61 log_10_ copies qPCR). A similar trend was shown in the control sample, where T_0h_ with a value of 1.34 log_10_ copies qPCR (*p* < 0.05) resulted significantly different compared to T_16h_ and T_24h_ with 0.36 and 0.32 log_10_ copies qPCR, respectively. At the same sampling time, *bsh* gene expression resulted more evident in the VB4-inoculated sample compared to the control one, which exhibited a low *bsh* gene expression due to the presence of commensal *L. rhamnosus* species.

For the *HKG* gene expression (Figure 3C), no significant differences (*p* > 0.05) were found between the VB4-inoculated sample at the sampling times, with a slight decrease from T_0h_ to T_24h_, with 6.94 to 6.33 log_10_ copies qPCR, respectively. Whereas in the control samples a significant difference was observed at all sampling times, with an increase from T_0h_ to T_24h_, with 1.99 and 3.24 log_10_ copies qPCR (*p* < 0.05), respectively. At the same sampling time, *HKG* gene expression confirmed the higher transcriptional ability of VB4-inoculated compared to control samples.

### 3.3. Semi-Quantitative Analysis of BAs in Small Intestine and Colon

In order to test whether the inoculum of *L. rhamnosus* VB4 strain changed the BAs profile, the amount of four conjugated BAs (taurocholic, glycocholic, taurodeoxycholic, and glycodeoxycholic acids), was detected by high-resolution mass spectrometry during small intestine and colon digestion.

**Figure 4:**
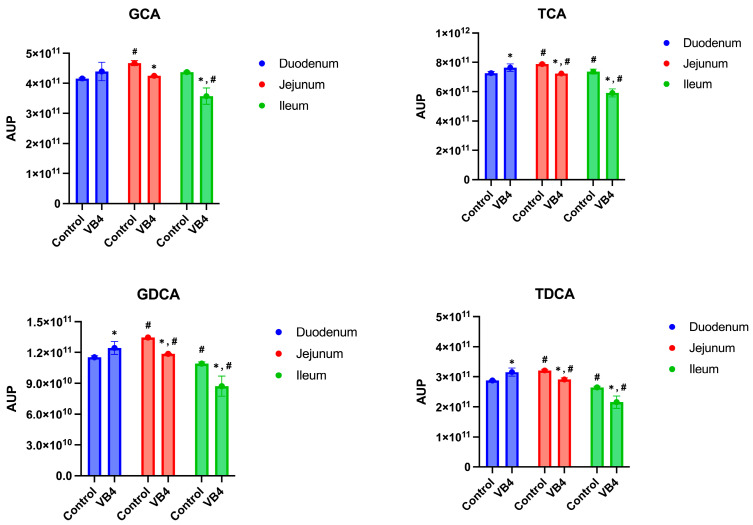
Conjugated BAs in the small intestine. Glycocholic acid (GCA); Taurocholic acid (TCA); Glycodeoxycholic acid (GDCA); Taurodeoxycholic acid (TDCA). Blue bars indicated duodenum samples, jejunum was identified by red bars, while green bars represent ileum. AUP means area under the peak and is an average of at least three replicates. Significant differences were established by two-way ANOVA using Dunnett’s post-hoc test (*p <* 0.05). * The superscript asterisks symbol indicates significant differences between inoculated and control samples at the same time at *p <* 0.05. ^#^ The hash symbol indicates significant differences within the same sample over time at *p <* 0.05. VB4, *L. rhamnosus* VB4 strain.

The amount of the conjugated BAs increased from the duodenum to the jejunum in the control vessels (Figure 4 and Table S1). Differently, a significant decrease was detected among the VB4-inoculated samples (*p* < 0.05), with the exception of the GCA sample, with values of 4.40 × 10^11^ ± 3.03 × 10^10^ AUP and 4.25 × 10^11^ ± 8.87 × 10^8^ AUP, in duodenum and jejunum, respectively (Figure 4 and Table S1). The amount of GCA, in control sample, was not affected after passing into the ileum (4.37 × 10^11^ ± 2.73 × 10^9^ AUP), whereas a significant (*p* < 0.05) decrease in the amount of the other conjugated acids was observed, while significant decrease (*p* < 0.05) was showed for the VB4-inoculated sample (Figure 4 and Table S1).

**Figure 5:**
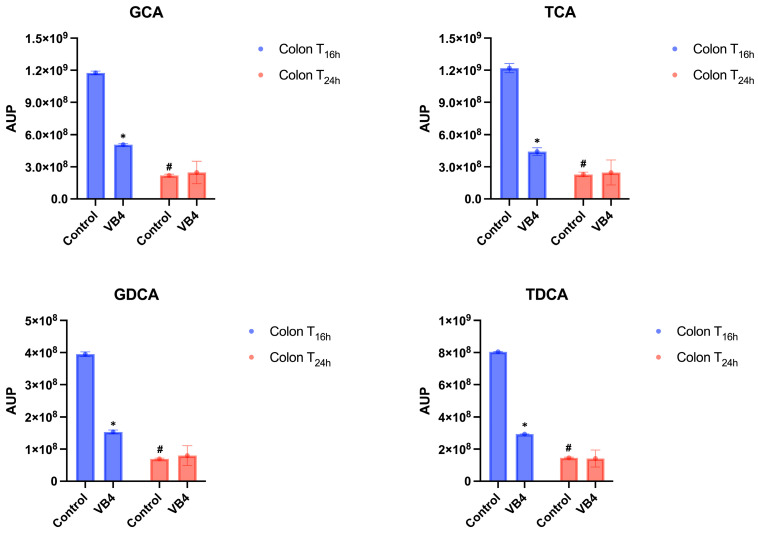
Conjugated BAs in the colon. Glycocholic acid (GCA); Taurocholic acid (TCA); Glycodeoxycholic acid (GDCA); Taurodeoxycholic acid (TDCA). Semi-transparent blue and red bars identified the different sampling time of colon samples, at 16 and 24 h, respectively. Two-way ANOVA using Dunnett’s post-hoc test was performed to assess the BAs profile changes after VB4 administration and BAs type. AUP means area under the peak (n = 3). * The superscript asterisks symbol indicates significant differences between inoculated and control samples at the same time at *p <* 0.05. ^#^ The hash symbol indicates significant differences within the same sample over time at *p <* 0.05. VB4, *L. rhamnosus* VB4 strain.

As expected, a decline of 2/3 orders of magnitude from ileum to colonic fraction were observed in both control and VB4-inoculates samples, probably due to the physiological dilution of digested during the transit in the colonic fraction, reaching a value of approximately of 10^9^–10^8^ and AUP in the colon fraction, after 16 h (T_16h_) of incubation (Figure 5 and Table S1). It is interesting to highlight that a more pronounced decrease of BAs was revealed in the VB4-inoculated samples, compared to the control. In the T_24h_ colon sample, no significant differences (*p* < 0.05) between the control and the inoculated conjugated BAs were found. Otherwise, in the VB4-inoculated reactor, the amount of conjugated BAs significantly decreased (*p* < 0.05) in T_24h_ colon samples compared with T_16h_ (Figure 5 and Table S1).

## 4. Discussion

Bile salt hydrolase (BSH) activity is a key probiotic function, particularly in *Lactobacillus* species, as it promotes gastrointestinal survival, modulates bile acid metabolism, and contributes to cholesterol reduction and cardiovascular health [17,18]. In the present study, BSH activity of the *L. rhamnosus* VB4 strain was assessed using the upper GIT SHIME^®^ model, under fed condition, providing a more realistic physiological state in which BSH activity can be optimally expressed as a probiotic feature. Furthermore, to elucidate the behavior of the VB4 administration under the influence of the gut microbial community, which represents a natural reservoir of BSH-positive bacteria in healthy individuals [19], the experiment was extended to the colonic fraction.

The strain survivability through the GIT passage was revealed by culture-dependent analysis and *bsh* gene expression profile was detected by qPCR. Across the simulated GI transit, the VB4 strain exhibited high resilience, with a modest loss of around 1 log unit, from the stomach to the ileum-end in fed condition. A similar upper GIT SHIME^®^ experiment was conducted by Govaert and collaborators [20], in which a freeze-dried probiotic formulation, inoculated at 9.7 log_10_ CFU/g under fasted (probiotic administration before a meal) condition, preserved high viability from the stomach to the ileal phases, showing around 2 log reduction in cell density at the final stage. According to that, previous studies have highlighted that exposure to gastric juice can drastically reduce probiotic survival. These findings figure out the ability of the probiotic strain to adapt to gastric pH and enzymes, as well as to bile salts and pancreatic enzymes, which represent an additional critical factor to be overcome.

According to transcriptional data, *bsh* gene expression showed significant variations in several compartments of the gastrointestinal tract. In particular, when inoculated, *L. rhamnosus* VB4 showed higher gene expression levels in the stomach and a gradual decline towards the duodenal, jejunal, and ileal phases.

Conversely, as observed in literature [21], that *bsh* gene expression is inducible in conditions of bile richness, in this study it is clear to see that its expression is already present and detectable in the gastric compartment and then attenuates with the decrease in bile salt concentrations along the small intestine. These data suggest that, applying the upper GIT SHIME^®^ system, in VB4 *bsh* transcription is detectable already in the gastric compartment (where no bile salts are present), consistent with a basal or ‘constitutive-like’ expression specific to this strain. In addition, *bsh* gene was further detected in the colon compartment up to 24 h of incubation. Despite the *bsh* expression being associated with the species *L. rhamnosus* as part of the commensal microbiota, VB4-inoculated samples displayed sustained *bsh* expression compared to the control sample, suggesting an overtime contribution of the VB4 strain in enhancing the deconjugation of BAs compared to the control.

Scientific evidence has revealed a close relationship between BAs deconjugation ability and *bsh* gene expression [9,22]. According to that, in this study the impact of the BAs profile after VB4 administration by detecting the main human glyco- and tauro-conjugated acids was demonstrated. Nevertheless, in human bile, conjugated bile acids are predominantly glycine- and taurine-conjugated forms, approximately with a ratio of 3:1, respectively. Total bile salt concentrations in human typically range from 20 to 40 mM [23]. Conversely, pancreatic juice for SHIME^®^ system included Oxgall (Difco), in which the taurine-conjugated fraction is proportionally higher than in human bile and the overall composition is less representative of the exact spectrum of human BAs [24]. Although Oxgall (bovine bile) is widely used in vitro to simulate the gastrointestinal environment of bile acids, these compositional differences must be taken into account for the comparison with human physiology.

In physiological conditions, BAs deconjugation starts in the small intestine with maximal activity occurring in the ileum and in the proximal colon [25,26]. On this basis, our study showed a progressive decrease in each detected conjugated BAs from the duodenum to the jejunum phases after VB4 administration, followed by a substantial decrease after the ileal phase. These results could confirm the highest deconjugation potential at the end of the small intestine. After 16 h of incubation (T16h), the colonic fraction with VB4 administration showed significantly lower levels of conjugated bile acids compared to the control. This reduction reflects both the prolonged effect of VB4-mediated deconjugation in the small intestine and the physiological dilution of digested intestinal contents in the colon, which was also revealed in the control reactor. However, at T_24h_, although the further reduction, VB4 did not alter BAs profile in the colonic fraction, except for a slight increase in GDCA (compared to the control). These finding suggests that, at this stage, VB4 is starting to lose its BSH potential, mainly expressed in the proximal colon stages, as described by Ridlon and co-worker [27] in their review article, which described the BAs metabolism.

## 5. Conclusions

This study provides novel insight into the dynamic interplay between the BSH-positve *L. rhamnosus* VB4 strain and BAs metabolism during the GIT. The obtained results demonstrate that VB4 exhibits robust gastrointestinal survivability and maintains transcriptional activity of the *bsh* gene, indicating its resilience during the harsh conditions of the digestive process. The modulation of the BAs profile, characterized by a marked reduction of conjugated BAs in the ileal phase, supports the strain’s active deconjugation potential. These findings contribute to the growing body of evidence to support the use of BSH-positive probiotics shaping host BAs pools. Combining untargeted metabolomics with dynamic gut models offers a valuable approach to study the probiotic influence on BAs biotransformation, posing a new possibility for developing a personalized probiotic treatment in restoring gut microbiota and BAs dysregulation. These approaches will enable future studies to be conducted focusing on the effect of a daily administration of BSH-positive strains on the gut microbiota, using hypercholesterolemic subjects, in order to clarify the positive and negative effects of BSH probiotic activity on host physiology.

## Data Availability

The original contributions presented in this study are included in the article. Further inquiries can be directed to the corresponding author.

## Supplementary Materials

The following supporting information can be downloaded at: https://www.mdpi.com/article/10.3390/nu17193179/s1, Table S1: Conjugated bile acids (BAs) detection in control and VB4-inoculated samples, under upper GIT SHIME® system. Glycocholic acid (GCA), taurocholic acid (TCA), glycodeoxycholic acid (GDCA), and taurodeoxycholic acid (TDCA) in duodenum, jejunum, ileum, and colon (T16h and T24h). Reported values are expressed as AUP ± SD.
