# Supplementary material for: Gastrointestinal Survivability of a BSH-Positive Lacticaseibacillus rhamnosus VB4 Strain and Its Effect on Bile Acid Deconjugation in a Dynamic In Vitro Gut Model
Source: Nutrients. 2025 Oct 8;17(19):3179. doi: 10.3390/nu17193179 (PMC12526281; doi:10.3390/nu17193179)
Supplement: Supplementary file 1 [file nutrients-17-03179-s001.zip › nutrients-3899632-supplementary.pdf]

Supplementary Tables

Gastrointestinal Survivability of a BSH-positive *Lacticaseibacillus rhamnosus* VB4 Strain and Its Effect on Bile Acid Deconjugation in a Dynamic *In Vitro* Gut Model

Amanda Vaccalluzzo[1†], Gianluigi Agolino[1†], Alessandra Pino[1,2], Marianna Cristofolini[3], Davide Tagliazucchi[3], Alice Cattivelli[3], Cinzia Caggia[1,2], Lisa Solieri[3], and Cinzia Lucia Randazzo[1,2,*]

[1] Department of Agriculture, Food and Environment, University of Catania, 95123 Catania, Italy; amanda.vaccalluzzo@unict.it (A.V.); gianluigi.agolino@phd.unict.it (G.A.); alessandra.pino@unict.it (A.P.); ccaggia@unict.it (C.G.); cinzia.randazzo@unict.it (C.R.)
[2] ProBioEtna srl, Spin Off of the University of Catania, Santa Sofia Street, 100, 95123 Catania, Italy; alessandra.pino@unict.it (A.P.); ccaggia@unict.it (C.G.); cinzia.randazzo@unict.it (C.R.)
[3] Department of Life Science, University of Modena and Reggio Emilia, 42122 Reggio Emilia, Italy; marianna.cristofolini@unimore.it (M.C.); davide.tagliazucchi@unimore.it (D.T.); alice.cattivelli@unimore.it (A. C.); lisa.solieri@unimore.it (L.S.)

* Correspondence: cinzia.randazzo@unict.it (C.L.R.)
† These authors contributed equally to this work

Table S1. Conjugated bile acids (BAs) detection in control and VB4-inoculated samples, under upper GIT SHIME® system. Glycocholic acid (GCA), taurocholic acid (TCA), glycodeoxycholic acid (GDCA), and taurodeoxycholic acid (TDCA) in duodenum, jejunum, ileum, and colon (T_{16h} and T_{24h}). Reported values are expressed as AUP ± SD.

Reactors	GCA		TCA		GDCA		TDCA	
	**Control**	**VB4**	**Control**	**VB4**	**Control**	**VB4**	**Control**	**VB4**
**Duodenum**	$4.16\times10^{11} \pm 2.61\times10^{9}$	$4.40\times10^{11} \pm 3.03\times10^{10}$	$7.26\times10^{11} \pm 1.20\times10^{10}$	$7.64\times10^{11} \pm 2.51\times10^{10}$	$1.15\times10^{11} \pm 1.49\times10^{9}$	$1.24\times10^{11} \pm 6.31\times10^{9}$	$2.88\times10^{11} \pm 1.27\times10^{9}$	$3.16\times10^{11} \pm 1.37\times10^{10}$
**Jejunum**	$4.67\times10^{11} \pm 8.38\times10^{9}$	$4.25\times10^{11} \pm 8.87\times10^{8}$	$7.88\times10^{11} \pm 7.17\times10^{9}$	$7.23\times10^{11} \pm 2.42\times10^{8}$	$1.35\times10^{11} \pm 0.00$	$1.19\times10^{11} \pm 3.22\times10^{8}$	$3.21\times10^{11} \pm 4.11\times10^{9}$	$2.91\times10^{11} \pm 5.16\times10^{9}$
**Ileum**	$4.37\times10^{11} \pm 2.73\times10^{9}$	$3.57\times10^{11} \pm 2.74\times10^{10}$	$7.37\times10^{11} \pm 1.64\times10^{10}$	$5.91\times10^{11} \pm 2.72\times10^{10}$	$1.09\times10^{11} \pm 1.70\times10^{9}$	$8.72\times10^{10} \pm 9.84\times10^{9}$	$2.65\times10^{11} \pm 3.94\times10^{9}$	$2.16\times10^{11} \pm 2.02\times10^{10}$
**Colon T_{16h}**	$1.18\times10^{9} \pm 1.60\times10^{7}$	$5.06\times10^{8} \pm 1.06\times10^{7}$	$1.22\times10^{9} \pm 4.24\times10^{7}$	$4.40\times10^{8} \pm 3.60\times10^{7}$	$3.95\times10^{8} \pm 7.25\times10^{6}$	$1.53\times10^{8} \pm 5.87\times10^{6}$	$8.04\times10^{8} \pm 1.77\times10^{6}$	$2.93\times10^{8} \pm 7.07\times10^{5}$
**Colon T_{24h}**	$2.20\times10^{8} \pm 1.21\times10^{7}$	$2.48\times10^{8} \pm 1.04\times10^{8}$	$2.29\times10^{8} \pm 2.13\times10^{7}$	$2.47\times10^{8} \pm 1.16\times10^{8}$	$7.00\times10^{7} \pm 2.55\times10^{6}$	$8.00\times10^{7} \pm 3.06\times10^{7}$	$1.46\times10^{8} \pm 3.85\times10^{6}$	$1.42\times10^{8} \pm 5.23\times10^{7}$
